# Endovascular Management of Uncontrollable Orbital Hemorrhage Secondary to Blast Injury

**DOI:** 10.7759/cureus.6713

**Published:** 2020-01-20

**Authors:** Megan Finneran, Michael Young, Hamad Farhat

**Affiliations:** 1 Neurosurgery, Advocate BroMenn Medical Center, Normal, USA; 2 Neurosurgery, Advocate Health Care, Oak Lawn, USA; 3 Neurosurgery, Advocate Christ Medical Center, Oak Lawn, USA

**Keywords:** ocular, blast injury, endovascular, onyx embolization, arterial hemorrhage, meningo-ophthalmic artery

## Abstract

Blast injuries to the face frequently involve vascular injury and have been reported in association with vehicles, including compressed air hoses and car battery explosions. While related to high-pressure releases, we present the first case of a car tire inflation resulting in tire explosion causing uncontrollable orbital hemorrhage, ocular damage, and the first case of endovascular intervention resulting in resolution of hemorrhage. A 63-year-old male presented after a tire explosion with evisceration of the right eye and uncontrollable hemorrhage from the orbit. CT demonstrated multiple maxillofacial fractures. Due to persistent hemorrhage, he was taken for emergent endovascular evaluation. On the angiogram, there was noted to be active extravasation from the right meningo-ophthalmic artery. Onyx® embolization of the right meningo-ophthalmic artery was performed with no further hemorrhage. Due to the severity of the injury, ophthalmology was unable to preserve vision in the eye. Arterial hemorrhages are traditionally managed with surgical exploration. However, endovascular management may be of particular utility in vascular injuries to the head and neck region. We highlight the importance of endovascular intervention to treat uncontrollable hemorrhage from orbit.

## Introduction

Ocular injury associated with blast injuries is a common occurrence [1]. The majority result from secondary blast events in which flying debris and fragments inflict the injury. The most common ocular consequences of blast injury include corneal abrasions, open globe injuries, eyelid lacerations, and intraocular foreign bodies [1]. There are multiple case reports on surgical management of ocular blast injuries [1-5] and further case reports of endovascular management for facial hemorrhages [6,7]; however, endovascular treatment for ocular hemorrhage has never been cited in the literature. We describe a case of ocular blast injury resulting from car tire inflation explosion that required emergent endovascular treatment of arterial hemorrhage.

## Case presentation

**Figure 1:**
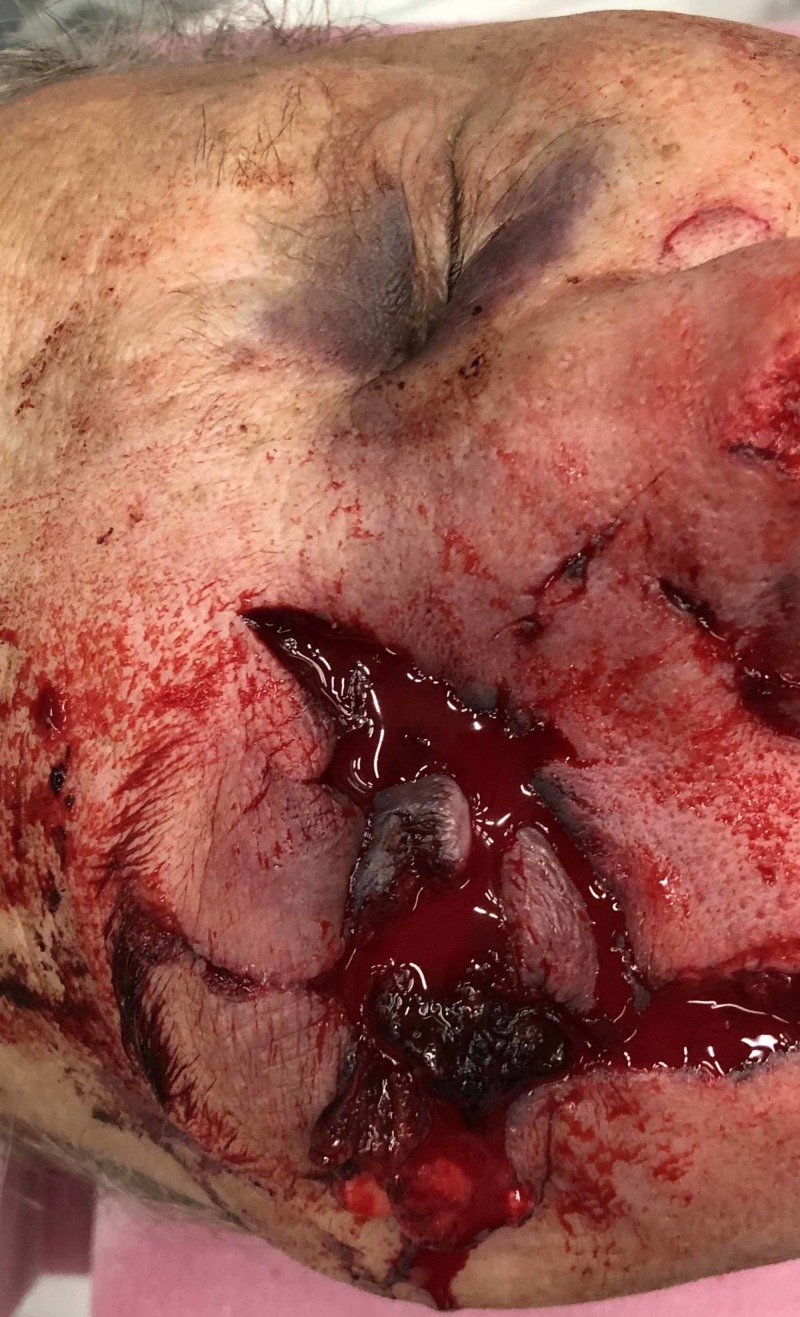
Uncontrollable arterial hemorrhage is observed from the right orbit with right globe rupture and evisceration of the right eye.

A 63-year-old-male presented to our level I trauma center after the car tire he was inflating exploded. Upon arrival, his airway was intact, he was breathing spontaneously, and he had good peripheral circulation. He sustained a right globe rupture and evisceration of the right eye with uncontrollable arterial hemorrhage from the orbit (Figure 1). 

**Figure 2:**
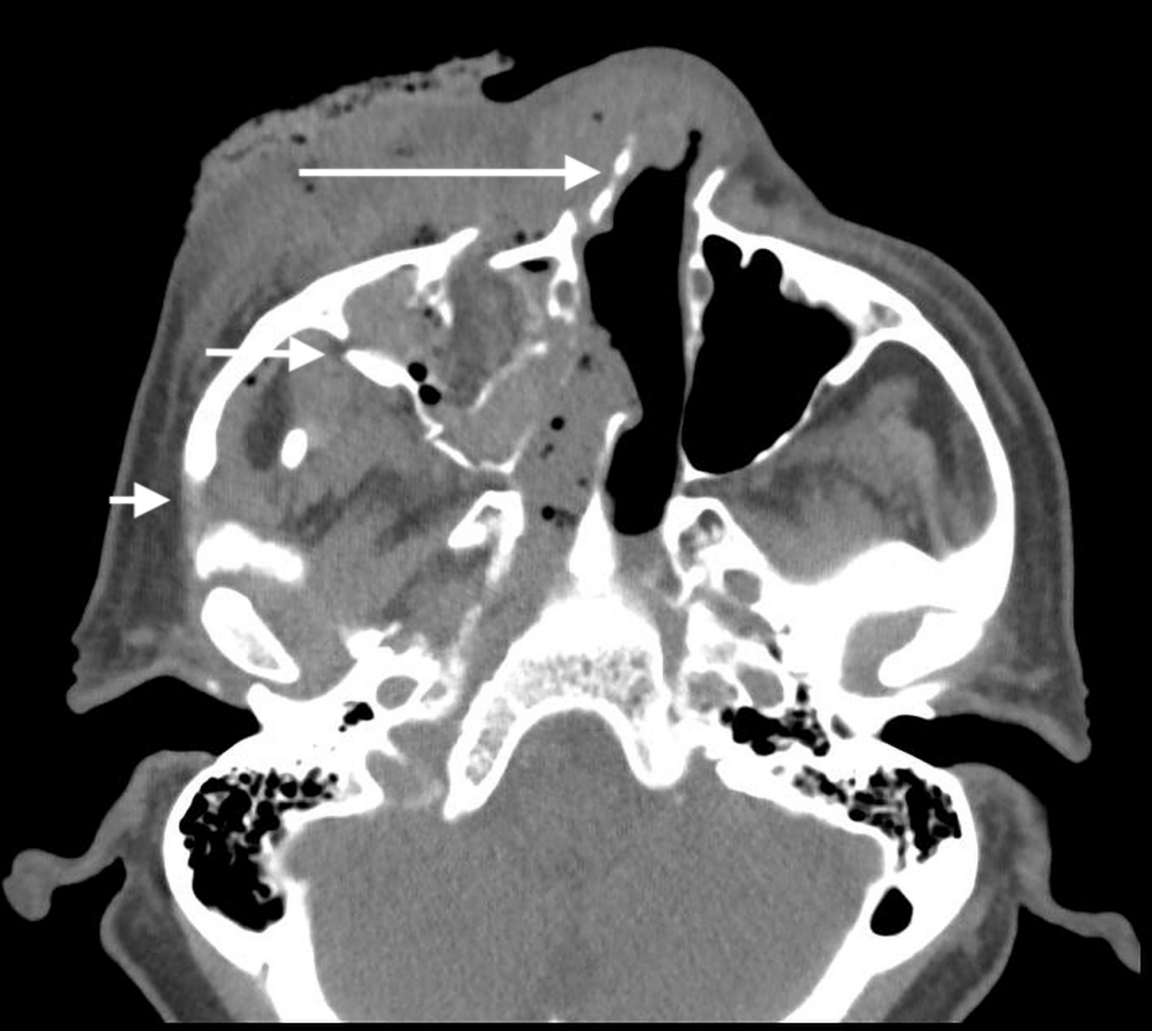
CT maxillofacial demonstrates fractures of the right zygoma (short arrow), lateral orbit (medium arrow), and nasal bones (long arrow).

CT maxillofacial showed fractures of the right zygoma, lateral orbit, and nasal bones (Figure 2). 

Head CT showed no evidence of intracranial injury. He presented without any neurologic deficits with the exception of blindness of the right eye.

**Figure 3:**
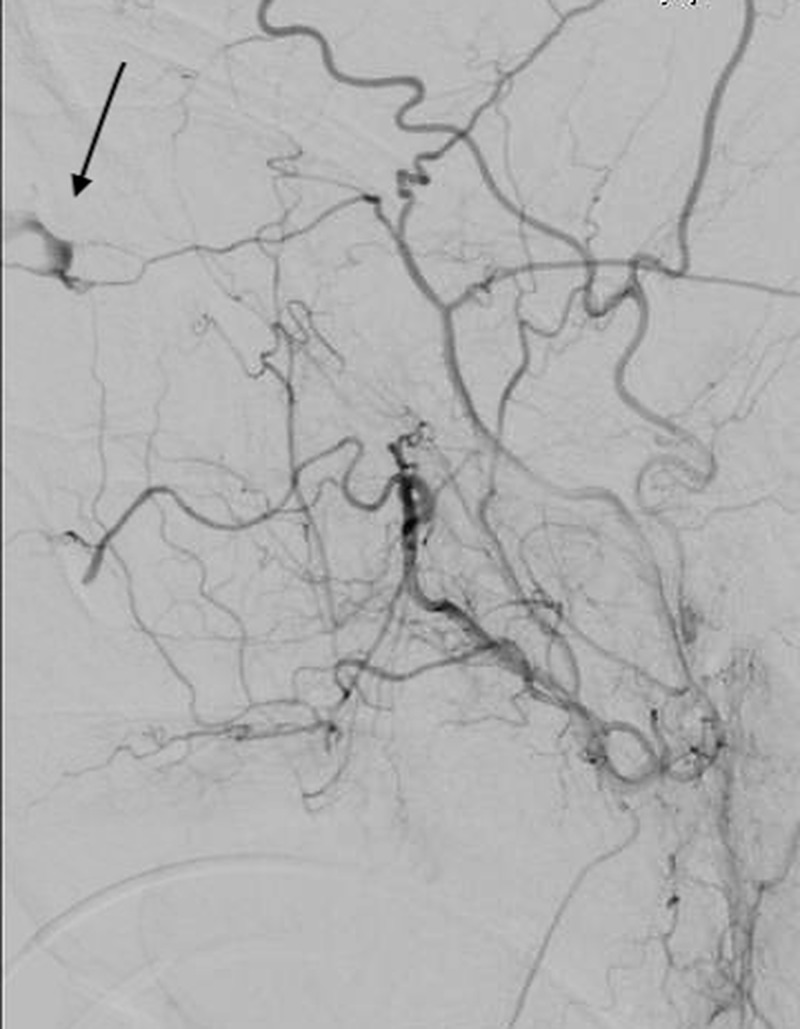
Lateral external carotid angiogram demonstrating active extravasation from the right meningo-ophthalmic artery.

Due to the uncontrolled arterial hemorrhage from the injury, he was taken emergently to the angiographic suite for possible embolization. Digital subtraction angiography of the right external carotid artery showed active extravasation of the right meningo-ophthalmic artery (Figure 3). 

**Figure 4:**
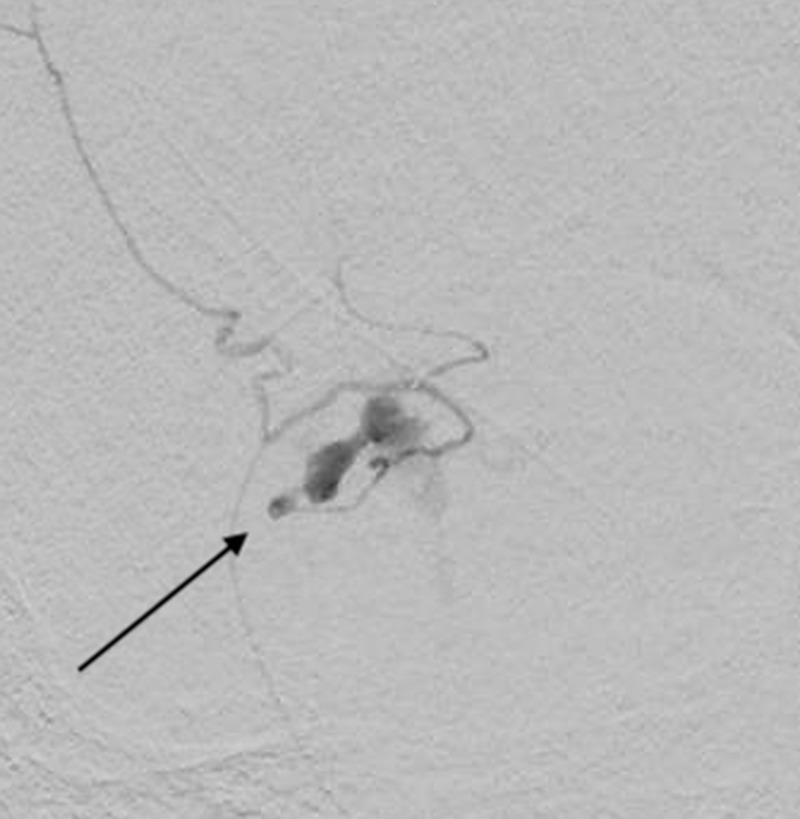
Superselective lateral angiogram showing microcatheterization of the right meningo-ophthalmic artery in preparation of embolization.

Superselective angiography was used to directly catheterize the hemorrhage from the right meningo-ophthalmic artery (Figure 4). 

**Figure 5:**
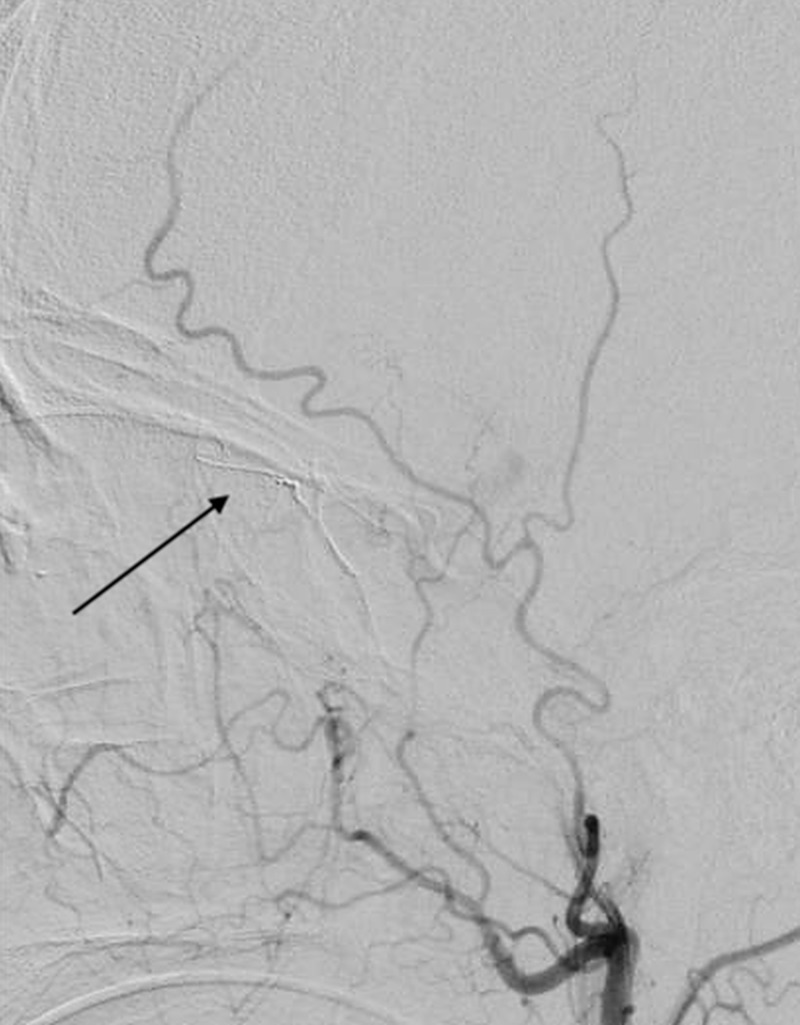
Lateral external carotid angiogram showing no further extravasation after Onyx embolization of the right meningo-ophthalmic artery.

**Figure 6:**
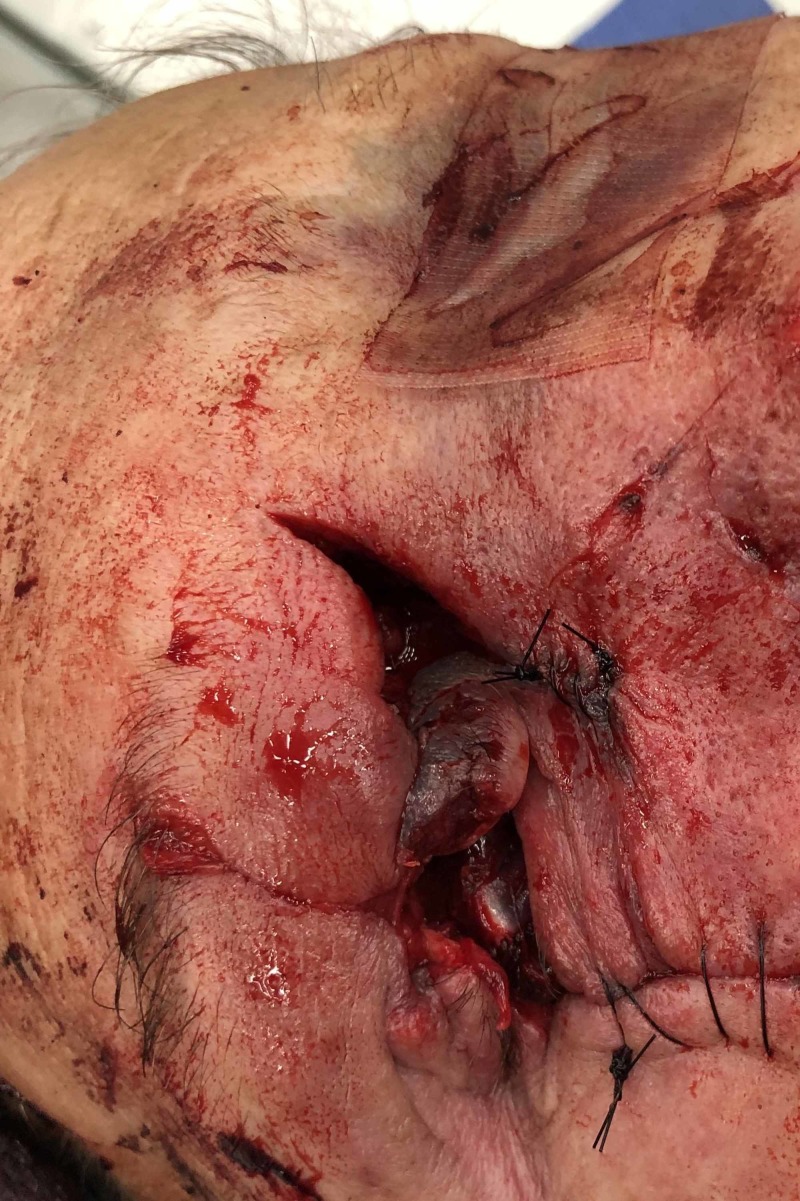
No further hemorrhage is visualized from the right orbit after Onyx embolization.

The right meningo-ophthalmic artery was embolized with Onyx® (ev3, Irvine, CA) without further extravasation (Figures 5, 6).

**Figure 7:**
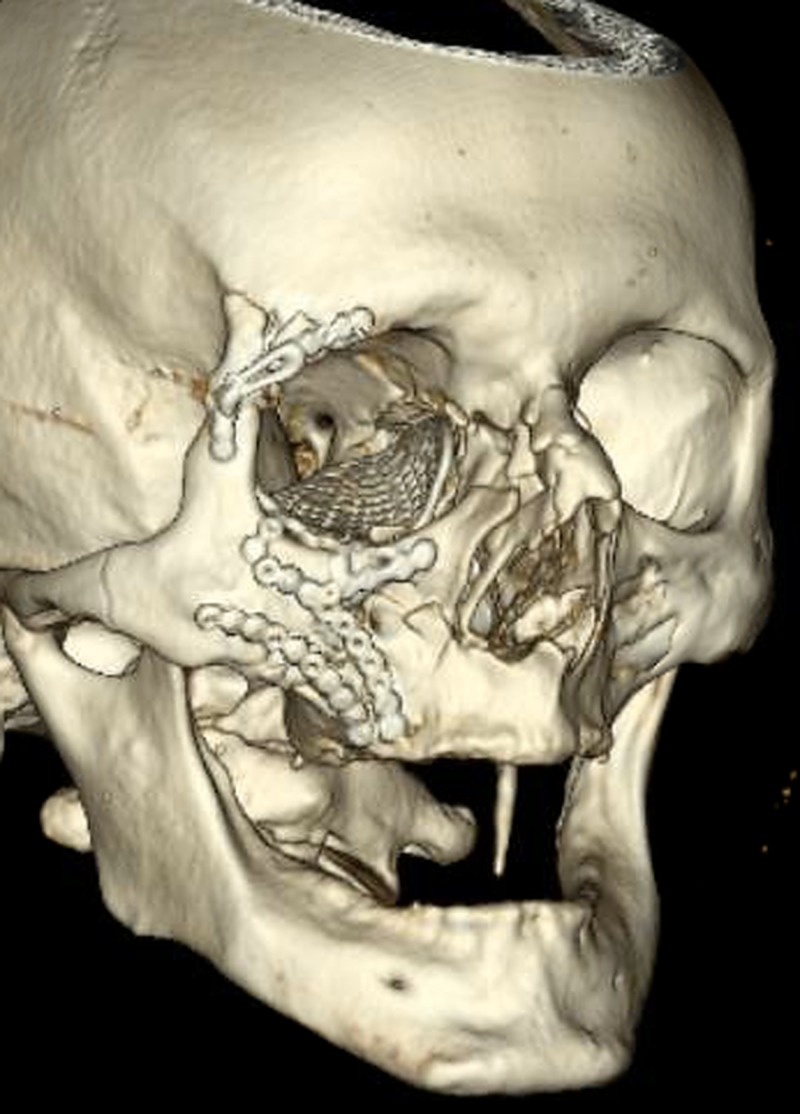
Three-dimensional maxillofacial reconstruction demonstrating open reduction and internal fixation of the complex zygomaticomaxillary complex fracture and orbital wall fractures.

The following day, the oculoplastic team performed open reduction and internal fixation of the zygomaticomaxillary complex fracture and orbital wall fractures with closed reduction of the nasal bone fractures (Figure 7). 

Ophthalmology was also consulted, but unfortunately was unable to preserve the eye. The patient remained neurologically intact after both procedures. He was discharged home on post injury day 5.

## Discussion

The management of arterial hemorrhage has traditionally relied on surgical exploration for arterial ligation. However, difficult exposure may limit the ability of direct visualization. A challenge is particularly posed in areas of the face, including the oral cavity, nasopharynx, and palatine fossa [8]. Additionally, attempts at proximal control may be complicated by extensive collateralization of the external carotid artery and difficult surgical exposure [6]. For these reasons, endovascular management of hemorrhagic head injuries is an increasingly common occurrence. 

While common for head injuries, endovascular treatment for ocular blast injuries has not been reported in the literature. Alam et al. described 79 patients with ocular injuries in blast victims [2]. Among these, treatment methods included medical management for 40 eyes and surgery for 68 eyes. The most common injuries cited included corneal/scleral perforation, vitreous hemorrhage, and traumatic cataract. Of all injuries, none were resolved with endovascular management. The authors commented that ophthalmologic evaluation is often delayed, as most ocular blast injuries take place in association with other potentially life-threatening injuries that may require hemodynamic and/or surgical stabilization. 

Multiple cases have been reported of car-related orbital injuries, but the majority of them are affiliated with car battery explosions [4,5]. Minatoya discussed 62 patients seen in a clinic setting for eye injuries from this cause, the majority of which were acid-induced conjunctivitis [4]. Moore et al. cited 10 serious injuries that were sequelae of car battery explosions [5]. Of these, the majority again resulted from battery acid. The three main mechanisms cited included chemical trauma, concussive damage from the acid, and traumatic damage from battery fragments or patient eyeglasses. In that study, none of the 10 patients wore safety goggles during the time of trauma. The authors commented that while no national or international data were available regarding statistics for battery-related eye injuries, it is well known that an increasing number of people in the general population are performing their own car maintenance. They carried out a public safety survey among car battery manufacturers to gather information of safety precautions shared with their products, finding they were often adequate but not prominently displayed. One conclusion from the article was the importance of safety procedures while conducting home vehicle maintenance. 

Hitchings and McGill described a case of an air hose emitting high-pressure air stream directed toward the eye from a few inches range, resulting in diplopia and orbital emphysema [3]. They discussed five possible consequences of high-pressure injury to the orbit: contusion, foreign body, inflammation due to infection or retained foreign body, displacement of orbital contents, and intracranial air traversing the optic nerve sheath or through an orbital wall fracture. In our case, the blast caused such severe displacement of the orbital contents that the eye was eviscerated on initial presentation. Despite the irreparable orbital damage, endovascular control of the presenting hemorrhage successfully stabilized the patient.

## Conclusions

Blast injuries to the face are fairly common and management often consists of surgical exploration and repair. Vascular injuries involving the head, including the orbit, may benefit from an endovascular approach. We present the first case reported in the literature of an ocular blast injury from a tire inflation explosion that required emergent endovascular embolization for arterial hemorrhage. This case offers an important treatment modality for a potentially devastating injury.
